# Gender Differences in Publication Output: Towards an Unbiased Metric of Research Performance

**DOI:** 10.1371/journal.pone.0000127

**Published:** 2006-12-27

**Authors:** Matthew R.E. Symonds, Neil J. Gemmell, Tamsin L. Braisher, Kylie L. Gorringe, Mark A. Elgar

**Affiliations:** 1 Department of Zoology, University of Melbourne, Victoria, Australia; 2 School of Biological Science, University of Canterbury, Christchurch, New Zealand; 3 Landcare Research, Lincoln, New Zealand; 4 Peter MacCallum Cancer Centre, Melbourne, Victoria, Australia; University of Exeter, Cornwall Campus, United Kingdom

## Abstract

We examined the publication records of a cohort of 168 life scientists in the field of ecology and evolutionary biology to assess gender differences in research performance. Clear discrepancies in publication rate between men and women appear very early in their careers and this has consequences for the subsequent citation of their work. We show that a recently proposed index designed to rank scientists fairly is in fact strongly biased against female researchers, and advocate a modified index to assess men and women on a more equitable basis.

## Introduction

The causes of differences in gender representation within the hierarchical structure of academic science remain contentious. In 2005 the flames of this controversy were fanned by the widely reported comments of Lawrence Summers [1] who argued that few females had progressed to the higher levels of scientific academia due to a general lack of an innate aptitude for science when compared to males, rather than the presence of any discrimination. This view has been strongly criticised by others [most recently Ben Barres in *Nature*, [2]], who argue that performance differences reflect discrimination against females, although support for this position is equivocal with investigations into gender bias in funding application success, for example, suggesting different conclusions [e.g. 3], [4]. The two arguments may not necessarily be exclusive because the scientific review process, whether for papers, funding or promotions, could be inherently biased towards traits, such as self-promotion and overt competitiveness, that may be more typically exhibited by males [5]. Theoretically, absolute metrics of research performance, based on a combination of quantity of research output and its quality or impact, should overcome these problems. Here we show that these metrics are also biased against female scientists, and propose a new metric to better assess research performance in the context of relative opportunity.

There is a clear difference between men and women in science with regard to the quantity of their research output. On average, males publish more papers than their female counterparts, a trend that is consistent across scientific disciplines and exists even when obvious mitigating factors are taken into consideration [6]–[9]. The causes of this difference are mysterious, hence the term ‘the productivity puzzle’ [6], [7]. A similar difference in number of scientific patents has also been recently documented [10]. Superficially, these data might support the ‘Summers hypothesis’ (so-called by Barres [2]), especially since no gender biases in manuscript assessment by journals have thus far been revealed [11]–[13]. However, it may also be a consequence of social factors. For example, women in faculty positions may be more greatly encumbered with extra non-research responsibilities as a result of their rarity and the desire to have a balance of males and females on administrative committees [14].

One explanation that may account for the productivity puzzle is that female researchers produce fewer but higher quality publications. For example, one survey of biochemists [15] found that females' publications were typically cited more than males' publications. If this hypothesis of quality versus quantity is correct, then it suggests that we should assess scientific ability by incorporating both of these aspects of research output.

## Materials and Methods

We examined whether a gender pattern of quality versus quantity holds for researchers in the field of evolutionary biology and ecology. The data were a subset of those used in a previous analysis [11], and comprised 39 female and 129 male researchers who hold research and faculty positions in the life sciences departments of British and Australian universities. All researchers are approximately of the same cohort, having started publishing scientific papers between 1990 and 1993. We followed their subsequent publication track record, as detailed on The Web of Science (Thomson Scientific USA, http://scientific.thomson.com/products/wos/), until the end of 2005, counting both the number of publications and the number of citations that each of those papers received. The data set is presented in the Supporting Information: Appendix A.

## Results and Discussion

**Figure 1:**
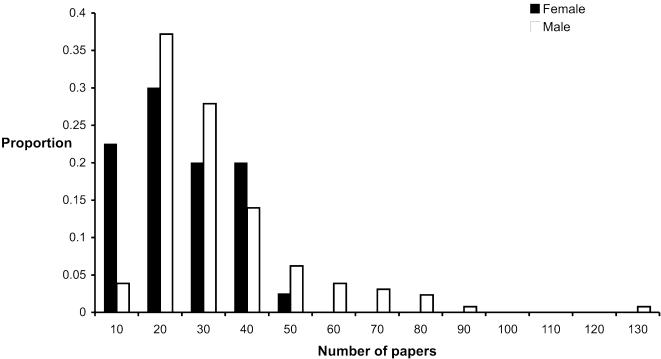
Frequency distributions of the number of publications by male and female researchers in our sample.

**Figure 2:**
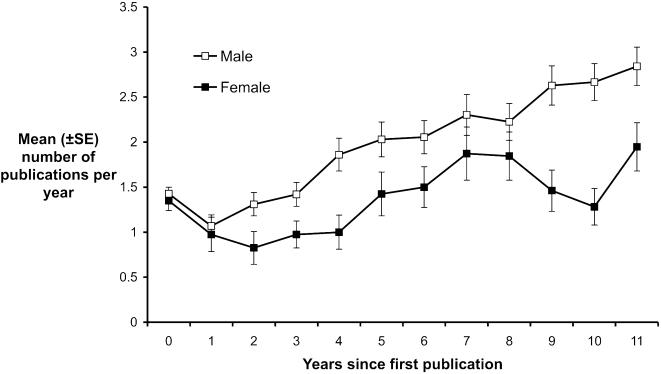
Annual productivity of male and female researchers over time.

Consistent with previous studies [9], [11], there is a clear difference in the number of publications produced by males and females in this field, with men publishing on average almost 40% more papers than women (mean number of publications = 28.26 and 20.23 respectively; t_102_ = −3.334, P = 0.001). The frequency distributions of numbers of publications for males and females also reveal differences (Fig. 1). Notably, there are proportionately very few males (<4%) with fewer than 10 publications, but almost a quarter (22.5%) of females fall into this category. In contrast, the higher end of the distribution drops off abruptly for females (there are none with more than 45 papers), whereas there is a long tail of a few hyper-productive males (14 with more than 50 publications). Differences in publication rates appear surprisingly early, with a clear discrepancy between males and females emerging 2 years after their first publication (Fig. 2). This is likely to correspond with the time just after doctoral thesis completion or during the first postdoctoral position. In fact, women take up to 5 years into their careers to achieve the same annual rate of output as men have at 2 years. The reason for this time delay is unknown, but we do not think it is indicative of a general inability of women to be productive: between years 4 and 8 the slope of the female productivity line parallels that of males (Fig. 2). If women were inherently less productive then the slope would be shallower for women than men. Whatever the reason behind this surprisingly early divergence in productivity, the pattern suggests that females might be in a situation where they are constantly ‘playing catch-up’ to their male colleagues throughout their career.

The graphs also indicate a second dip in productivity rates for females at around the 9–10 year mark. We can only speculate as to its cause, but it may coincide with a time when a number of factors have their greatest impact on female productivity, namely reduced success in grant rounds, time devoted to childcare, and greater administrative burden, as previously suggested. Many strategies implemented by universities to improve representation of women at higher levels in academia focus on mentoring programmes, with the intention of improving their competitiveness for funding, appointment and promotion. However, the implications of these productivity patterns are that, in most cases, such programmes may be offered too late to be useful. We suggest that such schemes need to be implemented at an extremely early career stage (i.e. at graduate student level).

Our analysis covers only researchers from one area of science, but an examination of gender differences in funding success across the arts and sciences suggest that these trends have broader generality. We examined age- and gender-specific success in the Australian Research Council's Discovery Grant awards over six years since 2001 (www.arc.gov.au). These grant applications cover all disciplines (except for clinical medicine) and are not confined to science. There is a clear discrepancy between the overall proportions of men and women being successful in grant applications (9,048 out of 31,511 = 28.7% for men vs. 2,388 out of 9,861 = 24.2% for women: χ^2^ = 75.945, df = 1, P<0.001). It is also worth noting that in 4 out of 6 years this gender-based discrepancy was greater for researchers under the age of 30. The two years where this was not the case were, perhaps not coincidentally, years where overall success rate in applications was high (see Table 1).

**Table 1:**
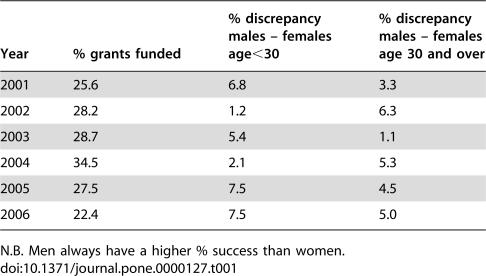
Discrepancies between male and female success rates in ARC grant applications 2001–2006 comparing junior scientists (aged under 30) with older scientists.

Year	% grants funded	% discrepancy males – females age<30	% discrepancy males – females age 30 and over
2001	25.6	6.8	3.3
2002	28.2	1.2	6.3
2003	28.7	5.4	1.1
2004	34.5	2.1	5.3
2005	27.5	7.5	4.5
2006	22.4	7.5	5.0

N.B. Men always have a higher % success than women.

There is no difference in the median number of citations per paper for males and females (median = 9 and 10 respectively; Mann-Whitney U = 2830.0, P = 0.237), which argues against a quality versus quantity hypothesis. Nor is there any evidence that men employ a more ‘hit and miss’ strategy for their output, with the variation in citations per paper being similar in males and females (median interquartile range = 15.50 and 13.75 respectively; U = 2653.5, P = 0.603). However, the first quartile of female median citations is significantly higher than that for males (median = 6 and 4 respectively; U = 3225.5, P = 0.007), indicating that there are relatively few females who produce a body of work that is poorly cited. Perhaps males who produce ‘poor quality’ work are more likely to survive in science than females.

However, drawing conclusions about the relationship between quantity and quality of research output is problematic if number of citations is used as the measure of quality because this metric is not independent of our measure of quantity. The median number of citations for our sample of authors is correlated with the number of papers they have published (r = 0.266, n = 168, P<0.001 – using log-transformed values). In other words, more-productive scientists produce more highly cited papers. Kelly & Jennions [9] previously speculated that this could be due to a ‘lottery effect’ such that researchers with more papers are more likely to have highly-cited papers by chance. Alternatively, researchers may proportionately over-cite papers by authors they most often encounter in the literature (a ‘fast-food effect’).

**Figure 3:**
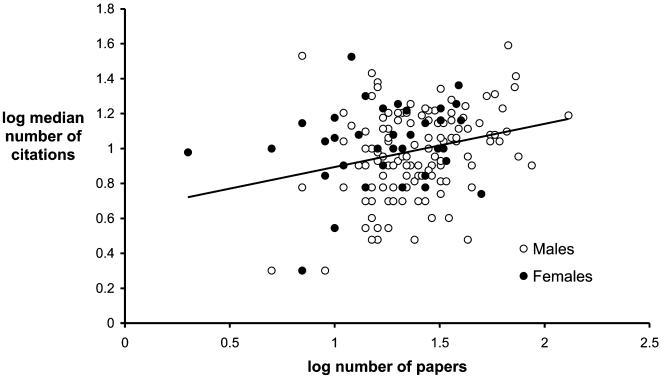
Relationship between quality of output (median number of citations) and quantity of output for male and female researchers.

We control for non-independence in our analysis by plotting the average number of citations per publication against total number of publications and calculating the y-residuals from the least squares regression line. When we do this (Fig. 3) we observe that female researchers tend to fall above the regression line indicating that they produce higher quality output than would be expected for their productivity, whereas males tend to be below the line (mean residual values = 0.07 and −0.02 respectively; t_65_ = 2.100, P = 0.041). In other words, for a given level of productivity, females produce better quality work than males. These data provide support for the idea that females produce higher quality research compared to their male counterparts, who tend to produce a greater quantity of research output.

One potential complicating factor that we have not considered is self-citation. Researchers are likely to cite their earlier publications to varying extents and this may be more likely if their body of output is larger. The rate of self-citation could influence our analysis if there are gender differences in the propensity to self-cite. We investigated this possibility using the Web of Science's ‘Citation Analysis Report’ option, which provides details of papers that have cited an author's work, with and without self-citations. We found no evidence of gender differences in the rates of self-citation, using a randomly chosen subset of 20 females and 20 males from our original sample (mean percentage of citing papers for an author that are by that author = 5.81% and 6.21% respectively; t_32_ = 0.310, P = 0.759). Accordingly, our broader analysis is unlikely to be systematically biased by any gender differences in the rates of self-citation.

Given that there are differences between males and females in the quantity, and potentially quality, of research output, how can we establish academic selection systems that do not discriminate on the grounds of gender? Clearly, criteria based solely on quantity of output would favour males, but our results show that even when quality of research is taken into account (through impact of papers) males may be favoured since this measure of quality is correlated with quantity. If we are to ensure that research performance is assessed without such gender bias, then we need a measure that takes into account the relationship between quality and quantity.

The recently proposed *h* index [16] is a new measure of research performance that has been heavily championed by *Nature* [17] and *Science* [18]. This measure is the number of papers, *h*, by a scientist where each paper has received *h* or more citations (ideally excluding self-citations [19]). We calculated *h* for our researchers, based on publications in the period 1996–2005 (thereby eliminating any effects of scientific age of the researcher). As previously noted [8], *h* is highly correlated with quantity of research output (r = 0.846, n = 168, P<0.001 in our sample), and thus female scientists assessed through this measure will also suffer in comparison with males.

We advocate an alternative metric to *h*, namely residual *h*, which we call *Research Status*. This value is calculated as the y-residual from the least-squares regression line of *h* on the number of publications. Calculation of *Research Status* requires data from a number of individuals in the same field. This would be feasible in the case of applications for competitive grants, where there may be several dozen or even hundreds of grants to assess, or indeed for the purposes of research assessment exercises. The applicants with the highest residual *h* would be those with the greatest proportion of their output that had significant impact. Such a measure would also control for effects of scientific age, which correlates with *h*, making calculation of *m* (*h* divided by age [16]) unnecessary. When we calculated research status for the scientists in our sample, we found no difference between males and females (mean residual *h* = −0.01 and 0.02 respectively; t_59_ = 1.054, P = 0.296).

While we believe that our new metric provides a more equitable measure of research performance, it is susceptible in a detrimental way to the addition of just a handful of poorly cited papers. This property might deter scientists from publishing minor works that contain essential but unexciting results. However, it is a moot point whether research that fails to make an impact is actually useful. An alternative view is that this metric might encourage scientists to think more carefully about the quality and potential impact of their research before embarking on a project.

A second problem with our *Research Status* metric is that it may appear to completely disregard the quantity of output. Thus, one researcher with a handful of papers will be judged equivalently to another with a substantial body of work. In fact, our metric takes into account the fact that *h* is expected to be proportionately higher for people with few publications (an *h* score of 4 with 5 publications is far more likely than an *h* of 40 with 50 publications), which mitigates this problem.

Clearly, an assessment of a scientific career should not ultimately boil down to a single number [9]. Nonetheless, our analysis illustrates the potential biases that exist within current research performance metrics. Our new metric provides a method for removing gender-based bias without recourse to socially divisive procedures such as setting different thresholds for men and women.

Of course, some will argue that shifting the means by which we assess scientific performance is artificial and undesirable. However, until the career structure of science finds ways to assess females and males on a level playing field that takes into account the prevalent gender differences and imbalances (whatever their causes), we will continue to perpetrate inequality, and fail to maximise our intellectual capital [20].

## Supporting Information

Appendix A Publication and citation information for the 168 researchers in our analysis.
(0.32 MB DOC)
