# Supplementary material for: Gender Differences in Publication Output: Towards an Unbiased Metric of Research Performance
Source: PLoS One. 2006 Dec 27;1(1):e127. doi: 10.1371/journal.pone.0000127 (PMC1762413; doi:10.1371/journal.pone.0000127)
Supplement: Appendix A — Publication and citation information for the 168 researchers in our analysis. (0.32 MB DOC) [file pone.0000127.s001.doc]

**Supporting Information: Appendix A**

Publication and citation information for the 168 researchers in our analysis. N.B. calculation of *h* is for papers published in the time period 1997-2006.

| **Male/Female** | **number of publications** | **median number of citations** | **1st quartile of citations** | **3^rd^ quartile of citations** | **h** |
| --- | --- | --- | --- | --- | --- |
| F | 10 | 11.5 | 6.25 | 18.25 | 6 |
| F | 9 | 7 | 5 | 18 | 5 |
| F | 31 | 10 | 6 | 26.5 | 10 |
| F | 14 | 6 | 2.5 | 10.5 | 5 |
| F | 34 | 8.5 | 4 | 21.25 | 10 |
| F | 12 | 33.5 | 16 | 46.5 | 7 |
| F | 19 | 10 | 4 | 21.5 | 9 |
| F | 13 | 12 | 4 | 16 | 7 |
| F | 10 | 15 | 11.5 | 18.5 | 7 |
| F | 27 | 14 | 7 | 67.5 | 9 |
| F | 27 | 6 | 3.5 | 23 | 7 |
| F | 7 | 2 | 0.5 | 3 | 2 |
| F | 5 | 10 | 5 | 14 | 3 |
| F | 27 | 7 | 3.5 | 11.5 | 9 |
| F | 21 | 10 | 7 | 26 | 10 |
| F | 38 | 18 | 7.25 | 37.5 | 18 |
| F | 21 | 6 | 1 | 13 | 4 |
| F | 10 | 3.5 | 2.25 | 7 | 3 |
| F | 27 | 7 | 3.5 | 13 | 9 |
| F | 20 | 18 | 6.75 | 30.25 | 12 |
| F | 22 | 16.5 | 10.5 | 36.25 | 9 |
| F | 50 | 5.5 | 2 | 10 | 10 |
| F | 7 | 14 | 12.5 | 20.5 | 5 |
| F | 14 | 20 | 13.25 | 24.75 | 9 |
| F | 32 | 17 | 9.75 | 55 | 12 |
| F | 39 | 23 | 8 | 49 | 15 |
| F | 16 | 10 | 7.75 | 21.5 | 9 |
| F | 17 | 8 | 6 | 13 | 6 |
| F | 11 | 8 | 2 | 23 | 3 |
| F | 40 | 14.5 | 7 | 29.25 | 12 |
| F | 2 | 9.5 | 7.75 | 11.25 | N/A |
| F | 19 | 12 | 2.75 | 18.25 | 5 |
| F | 17 | 17 | 11 | 29 | 9 |
| F | 32 | 14.5 | 8 | 33.25 | 13 |
| F | 9 | 11 | 4 | 18 | 5 |
| F | 23 | 12 | 4 | 15 | 9 |
| F | 13 | 12 | 10 | 20 | 7 |
| F | 33 | 10 | 6 | 19 | 12 |
| F | 11 | 8 | 3.5 | 38.5 | 5 |
| M | 42 | 17.5 | 6 | 33 | 19 |
| M | 24 | 6.5 | 2.5 | 13.25 | 8 |
| M | 32 | 22 | 3 | 63.25 | 10 |
| M | 55 | 12 | 6 | 40.5 | 21 |
| M | 15 | 6 | 3 | 9 | 6 |
| M | 16 | 24 | 8.5 | 36.75 | 7 |
| M | 58 | 20.5 | 9 | 53.75 | 21 |
| M | 23 | 8 | 5 | 19.75 | 9 |
| M | 19 | 6 | 2 | 17.5 | 6 |
| M | 20 | 14.5 | 2.75 | 31 | 7 |
| M | 32 | 6.5 | 2 | 17.5 | 10 |
| M | 14 | 3.5 | 2 | 6 | 5 |
| M | 9 | 2 | 0 | 5 | 3 |
| M | 33 | 11 | 7 | 36 | 11 |
| M | 18 | 3.5 | 1 | 8.75 | 4 |
| M | 43 | 9 | 3.5 | 20 | 15 |
| M | 23 | 12 | 7 | 15 | 8 |
| M | 36 | 19 | 4 | 52.5 | 16 |
| M | 18 | 5 | 3.25 | 14 | 7 |
| M | 21 | 16 | 4 | 24 | 10 |
| M | 73 | 26 | 9 | 55 | 28 |
| M | 22 | 7 | 2.25 | 21 | 7 |
| M | 15 | 20 | 6.5 | 46 | 8 |
| M | 16 | 9.5 | 6.5 | 18.25 | 8 |
| M | 32 | 8 | 3 | 12 | 9 |
| M | 25 | 8 | 4 | 14 | 10 |
| M | 20 | 8.5 | 1.75 | 17.25 | 8 |
| M | 31 | 14 | 6 | 33.5 | 14 |
| M | 17 | 15 | 9 | 26 | 10 |
| M | 19 | 6 | 4 | 10.5 | 7 |
| M | 87 | 8 | 4 | 13 | 14 |
| M | 14 | 8 | 4.25 | 14 | 7 |
| M | 7 | 34 | 18 | 49.5 | 3 |
| M | 15 | 4 | 2 | 5 | 4 |
| M | 15 | 10 | 2 | 33.5 | 4 |
| M | 25 | 5 | 3 | 13 | 7 |
| M | 13 | 8 | 7 | 11 | 8 |
| M | 23 | 18 | 10 | 25.5 | 12 |
| M | 66 | 12.5 | 5.25 | 26.75 | 18 |
| M | 18 | 5 | 1.25 | 15.75 | 5 |
| M | 34 | 6.5 | 3.25 | 13 | 8 |
| M | 30 | 9 | 4.5 | 17.75 | 9 |
| M | 16 | 3 | 1 | 6 | 4 |
| M | 24 | 3 | 2 | 8 | 5 |
| M | 17 | 6 | 3 | 13 | 7 |
| M | 29 | 8 | 4 | 17 | 11 |
| M | 45 | 6 | 2 | 21 | 10 |
| M | 12 | 13.5 | 3.75 | 32.5 | 6 |
| M | 53 | 20 | 9 | 40 | 21 |
| M | 30 | 16.5 | 9.25 | 25.25 | 14 |
| M | 58 | 12 | 5 | 23.5 | 14 |
| M | 43 | 13 | 6.5 | 28 | 16 |
| M | 14 | 3.5 | 2 | 10.75 | 5 |
| M | 14 | 12.5 | 3.5 | 18 | 6 |
| M | 38 | 11 | 4 | 23.75 | 13 |
| M | 18 | 13 | 9 | 23.5 | 9 |
| M | 16 | 8.5 | 4.75 | 22.25 | 8 |
| M | 29 | 4 | 1 | 7 | 7 |
| M | 28 | 11.5 | 6 | 17.75 | 11 |
| M | 28 | 16.5 | 9.5 | 25.75 | 13 |
| M | 36 | 11.5 | 5.5 | 19.25 | 14 |
| M | 21 | 9 | 3 | 31 | 10 |
| M | 15 | 3 | 2 | 6 | 5 |
| M | 15 | 27 | 14.5 | 37 | 11 |
| M | 16 | 3.5 | 1 | 12.25 | 4 |
| M | 49 | 14 | 5 | 28 | 16 |
| M | 18 | 8 | 2.5 | 23.75 | 7 |
| M | 25 | 7 | 3 | 12 | 7 |
| M | 130 | 15.5 | 4.25 | 29.5 | 28 |
| M | 33 | 14 | 5 | 32 | 13 |
| M | 32 | 5.5 | 2 | 14.25 | 8 |
| M | 26 | 15.5 | 9 | 31 | 13 |
| M | 14 | 12.5 | 3 | 16.75 | 7 |
| M | 23 | 14 | 5 | 19 | 9 |
| M | 41 | 15 | 7 | 46 | 14 |
| M | 27 | 17 | 12.5 | 31 | 15 |
| M | 20 | 16 | 5.5 | 30 | 10 |
| M | 19 | 8 | 4.5 | 16.5 | 9 |
| M | 55 | 11 | 5 | 21 | 18 |
| M | 5 | 2 | 0 | 2 | 1 |
| M | 7 | 6 | 2.5 | 8.5 | 3 |
| M | 19 | 5 | 2.5 | 11 | 7 |
| M | 11 | 8 | 3.5 | 23.5 | 5 |
| M | 36 | 16 | 5 | 30.25 | 13 |
| M | 35 | 4 | 2 | 12 | 7 |
| M | 15 | 5 | 2.5 | 11.5 | 4 |
| M | 75 | 9 | 3 | 23 | 19 |
| M | 28 | 10 | 5 | 16.5 | 10 |
| M | 19 | 10 | 5 | 16 | 8 |
| M | 18 | 16 | 2.75 | 30.5 | 8 |
| M | 32 | 8.5 | 5 | 23 | 13 |
| M | 43 | 3 | 2 | 5.5 | 7 |
| M | 26 | 7 | 5 | 13.75 | 10 |
| M | 21 | 10 | 4 | 20 | 7 |
| M | 11 | 16 | 6 | 27.25 | 5 |
| M | 18 | 11 | 3.25 | 23.75 | 4 |
| M | 27 | 5 | 3 | 15 | 6 |
| M | 61 | 11 | 3 | 31 | 16 |
| M | 63 | 17 | 5.5 | 47 | 19 |
| M | 22 | 6 | 2 | 28.75 | 6 |
| M | 14 | 5 | 2.25 | 7.5 | 5 |
| M | 29 | 8 | 3 | 19 | 8 |
| M | 14 | 10 | 6.25 | 13.75 | 7 |
| M | 72 | 22.5 | 6.75 | 36 | 26 |
| M | 16 | 22.5 | 11.5 | 44 | 9 |
| M | 11 | 6 | 3.5 | 13 | 4 |
| M | 23 | 8 | 3.5 | 15.5 | 10 |
| M | 28 | 7.5 | 4.75 | 14.25 | 6 |
| M | 29 | 7 | 2 | 12 | 9 |
| M | 28 | 14.5 | 5 | 29.5 | 8 |
| M | 18 | 11.5 | 4.25 | 23 | 7 |
| M | 21 | 5 | 1 | 21 | 7 |
| M | 23 | 8 | 5 | 14 | 8 |
| M | 39 | 13 | 7 | 23.5 | 15 |
| M | 38 | 11.5 | 5 | 19.75 | 10 |
| M | 17 | 13 | 4 | 31 | 8 |
| M | 17 | 7 | 3 | 20 | 5 |
| M | 36 | 14.5 | 5 | 30.25 | 14 |
| M | 24 | 10.5 | 4.75 | 22.5 | 10 |
| M | 32 | 11.5 | 6.75 | 16.25 | 12 |
| M | 9 | 7 | 5 | 16 | 4 |
| M | 11 | 11 | 8.5 | 18 | 7 |
| M | 15 | 6 | 2 | 9 | 5 |
| M | 17 | 9 | 3 | 33 | 9 |
| M | 22 | 9 | 3 | 13 | 10 |
| M | 22 | 8 | 3.25 | 23 | 6 |
| M | 45 | 8 | 2 | 16 | 13 |
| M | 22 | 16 | 9.25 | 41.5 | 11 |
| M | 67 | 39 | 17.5 | 120 | 33 |
